# Special Issue “Adenosine Receptors in Health and Disease”

**DOI:** 10.3390/ijms252212370

**Published:** 2024-11-18

**Authors:** Srdjan M. Vlajkovic

**Affiliations:** 1 Department of Physiology, Faculty of Medical and Health Sciences, The University of Auckland, Private Bag 92019, Auckland 1142, New Zealand; s.vlajkovic@auckland.ac.nz; 2 Eisdell Moore Centre, Faculty of Medical and Health Sciences, The University of Auckland, Private Bag 92019, Auckland 1142, New Zealand

Adenosine receptor signalling regulates various physiological processes throughout the body. G protein-coupled adenosine receptors (A_1_, A_2A_, A_2B_ and A_3_) are broadly distributed, with exceptionally high expression levels in the central nervous, immune, and cardiovascular systems. Diverse physiological roles of adenosine receptors (ARs) include neuroprotection, immune modulation, regulation of cardiovascular function, and sleep. Adenosine acts as a cytoprotective modulator in response to stress and tissue injury; thus, understanding AR function is crucial for developing targeted therapies for many diseases [1].

Dysregulation of AR signalling can lead to various pathologies, such as pain, cancer, neurodegenerative, inflammatory, and autoimmune diseases. For example, AR malfunction is associated with neurological disorders such as epilepsy and Parkinson’s disease. In inflammatory conditions, AR modulation can either suppress or exacerbate immune responses, which is context- and organ-specific. AR-targeted therapies are being explored for various conditions, including wound healing, asthma, inflammatory bowel disease, rheumatoid arthritis, and neurodegenerative disorders [2].

Addressing the diversity of physiological and pathophysiological functions of adenosine receptors is a central feature of this Special Issue. We invited investigators to contribute original research articles and state-of-the-art reviews addressing the various roles of ARs in health and disease. The collection includes two original research papers and four review articles from international groups investigating different aspects of AR signalling. These articles are briefly summarised here.

Adenosine A_2A_ receptors (A_2A_Rs) represent a promising non-dopaminergic target for treating Parkinson’s disease (PD), with potential benefits extending beyond motor symptom management to neuroprotection and improvement of non-motor symptoms. Istradefylline, an A_2A_ receptor antagonist, was approved by the US Food and Drug Administration (FDA) in 2019 as an adjunct to levodopa for managing “off” episodes in PD when the symptoms return between medication doses. In a rat model of PD, Nunes et al. [3] demonstrated more substantial benefits of A_2A_R antagonists at the earlier stages of PD than after apparent neurodegeneration. PD was induced in rats by intracerebroventricular infusion of a neurotoxin 1-Methyl-4-phenylpyridinium (MPP^+^). MPP^+^ induces a slowly evolving neurodegeneration, with a temporal separation between the initial synaptotoxicity in the striatum and the delayed degeneration of dopaminergic neurons. Daily intraperitoneal injections of the A_2A_ receptor antagonist SCH58261 for 10 days showed a dual benefit, preventing both early striatal synaptotoxicity and delayed neurotoxicity in the substantia nigra (SN). However, the A_2A_R inhibition demonstrated a more robust behavioural improvement by preventing early striatal synaptotoxicity than delayed SN neurotoxicity. This preclinical study shows that A_2A_R antagonists are more effective at disease onset than later stages.

Kuczeriszka and Dobrowolski [4] compared the role of adenosine and nitric oxide (NO) systems in the regulation of renal function in female normoglycaemic (NG) and streptozotocin-induced diabetic rats (DM). Their study identified some interesting differences between male and female rats. Unlike male rats, hyperglycaemia did not alter the vasoactive influence of the adenosine system in the kidneys of female rats. Enhanced vasodilator influence of NO was observed in diabetic female rats, which was absent in previous studies on diabetic males. AR inhibition had little impact on NO levels in the kidney tissues of female rats, in contrast to a distinct increase reported in males. In DM female rats, lowered baseline renal excretion was observed due to the prevalence of the anti-natriuretic action of adenosine A_1_ receptors over natriuretic A_2_ receptors. Interestingly, an opposite receptor balance pattern was previously observed in male rats. The study thus revealed sex-related differences in adenosine and NO systems in both NG and DM rats and suggested that the balance of A_1_ and A_2_ receptors in DM rats can distinctly differ between males and females. Sex-related differences observed in this preclinical study may have significant clinical implications for the treatment of diabetic patients.

Ehlen et al. [5] selected and reviewed 29 studies investigating the use of indirect AR agonists in bone regeneration. AR activation, especially A_2A_R stimulation, improves bone regenerative potential by osteoblast stimulation and osteoclast inhibition. Indirect AR agonists have emerged as a promising approach for promoting bone regeneration and healing. This approach involves increasing endogenous adenosine levels or using compounds that indirectly activate ARs to enhance bone formation. Potential therapeutic tools include dipyridamole (adenosine transport inhibitor), ticagrelor (P2Y_12_ receptor antagonist and adenosine transport inhibitor), other inhibitors of the equilibrative nucleoside transporter 1 (ENT-1), CD39 and CD73 agonists promoting extracellular ATP and AMP hydrolysis to adenosine, adenosine deaminase inhibitors, and adenosine kinase inhibitors. The review discussed the mechanisms of each indirect AR agonist, their cellular responses, and their effects on bone formation. This review underlines the significant potential of indirect AR agonists as a novel therapeutic strategy for treating bone defects and enhancing fracture healing. The authors also reviewed controlled release technologies (nanofibers, microgels, nanoparticles, and liposomes) that provide sustained adenosine release at the bone defect site, thus enhancing bone regeneration and reducing systemic side effects.

Inflammatory skin diseases like psoriasis and contact hypersensitivity involve complex interactions among immune cells, cytokines, and other mediators. ARs are implicated in the pathogenesis of skin conditions through their ability to modulate inflammation and immune responses. AR targeting can potentially suppress inflammation and the remodelling of the epidermal structure. Chen et al. [6] extensively reviewed the role of AR agonists and antagonists in inflammatory skin conditions such as psoriasis, eczema, and allergic contact dermatitis. Positive outcomes achieved by using A_2A_R and A_3_R agonists in animal models and clinical trials underscore a promising therapeutic approach for managing inflammatory skin disorders. A_2A_Rs are particularly important in inhibiting inflammatory processes and promoting wound healing. Preclinical studies have shown that activating A_2A_Rs with agonists such as CGS 21680 can reduce epidermal hyperplasia and skin inflammation without causing side effects (e.g., skin atrophy). The A_3_R agonists have shown promise in reducing chronic inflammation in conditions like psoriasis. This review also points out that adenosine may have pro- and anti-inflammatory effects, depending on the receptor pattern expressed by keratinocytes. However, the topical treatment of inflammatory skin conditions can preclude systemic side effects of AR ligands, which can be utilised in future preclinical and clinical studies.

A review by Marchi et al. [7] focuses on the role of adenosine in the diagnosis and treatment of coronary artery disease (CAD). While not a primary treatment, the cardioprotective effects of adenosine have been studied in the context of acute myocardial infarction, with potential benefits in reducing infarction size. It is well established that ARs affect vasomotor function, blood pressure, heart rate, and platelet activity. Cardiovascular effects are receptor-specific; for example, A_1_ and A_3_ receptor activation protects against ischeamia–reperfusion injury, while coronary vasodilation and anti-inflammatory effects are primarily mediated via A_2A_ receptors. Adenosine, dipyridamole, and regadenoson are widely used as coronary vasodilators during cardiac stress imaging for CAD diagnosis. The authors also suggest that adenosine signalling pathways may be critical in regulating cardiac remodelling processes, including fibroblast activation, extracellular matrix deposition, and inflammation. Ongoing research is focused on the discovery of more selective AR agonists that could provide targeted therapies with limited side effects. The role of adenosine in long-term cardioprotection and management of chronic CAD is also an area of active investigation. In conclusion, adenosine plays a multifaceted role in interventional cardiology, serving as a diagnostic tool and a potential therapeutic agent in CAD.

The adenosine A_3_ receptor (A_3_R) has emerged as a significant player in cardiovascular health, particularly in the context of heart diseases. Duangrat et al. [8] reviewed signalling pathways associated with A_3_R and the potential therapeutic benefits of A_3_R agonists in cardiovascular disorders. A_3_R activation contributes to reduced cardiac damage in conditions such as ischaemic heart disease, heart failure, and hypertension by activating pro-survival signalling pathways such as extracellular signal-regulated kinase 1/2 (ERK1/2) and phosphoinositide 3-kinase (PI3K)/Akt. Activation of these pathways reduces infarction size and protects heart tissue during ischaemia/reperfusion injury. The A_3_R also exerts anti-inflammatory actions that minimise further damage to heart tissue. Compounds targeting A_3_R have shown promise in preclinical studies for their cardioprotective effects without significant adverse outcomes. Several A_3_R agonists, such as piclidenoson (IB-MECA) and namodenoson (2Cl-IB-MECA), exert cardioprotective effects against ischaemia in various animal models of heart disease. These therapies could potentially ameliorate cardiovascular complications by altering the expression and activity of A_3_R. However, species differences in A_3_R-mediated effects should be considered when translating findings from animal models to humans. There are also disparities in adenosine binding affinity of the A_3_R between humans and rodent models. The authors suggest that more preclinical and clinical research is required to optimise A_3_R-based therapeutic strategies for heart diseases.

This collection of original and review articles presents further evidence that ARs play vital roles in health and disease, offering potential therapeutic targets for various pathologies. Continued research in this field may lead to novel AR-based therapies to enhance bone regeneration and improve treatments for diabetes, Parkinson’s disease, and renal, skin, and cardiovascular disorders. While ARs represent promising therapeutic targets, several challenges remain, particularly attaining receptor subtype selectivity and minimising side effects due to the widespread AR distribution. We also need to understand the complex interplay between AR subtypes, often showing antagonistic effects upon stimulation. Future research will focus on developing more selective ligands and exploring combination therapies to maximise therapeutic benefits while minimising adverse side effects. Integrating novel therapy strategies into controlled clinical studies will permit significant advances in this research field and lead to novel and exciting health outcomes.

## References

[1] Haskó G., Linden J., Cronstein B., Pacher P. (2008). Adenosine receptors: Therapeutic aspects for inflammatory and immune diseases. Nat. Rev. Drug Discov..

[2] Jacobson K., Gao Z.-G. (2006). Adenosine receptors as therapeutic targets. Nat. Rev. Drug Discov..

[3] Nunes A.C.L., Carmo M., Behrenswerth A., Canas P.M., Agostinho P., Cunha R.A. (2024). Adenosine A_2A_ receptor blockade provides more effective benefits at the onset rather than after overt neurodegeneration in a rat model of Parkinson’s disease. Int. J. Mol. Sci..

[4] Kuczeriszka M., Dobrowolski L. (2024). Sex dependence in control of renal haemodynamics and excretion in streptozotocin diabetic rats—Role of adenosine system and nitric oxide. Int. J. Mol. Sci..

[5] Ehlen Q.T., Mirsky N.A., Slavin B.V., Parra M., Nayak V.V., Cronstein B., Witek L., Coelho P.G. (2024). Translational experimental basis of indirect adenosine receptor agonist stimulation for bone regeneration: A review. Int. J. Mol. Sci..

[6] Chen L., Lei X., Mahnke K. (2024). Adenosine and its receptors in the pathogenesis and treatment of inflammatory skin diseases. Int. J. Mol. Sci..

[7] Marchi E., Muraca I., Berteotti M., Gori A.M., Valenti R., Marcucci R. (2024). Adenosine in interventional cardiology: Physiopathologic and pharmacologic effects in coronary artery disease. Int. J. Mol. Sci..

[8] Duangrat R., Parichatikanond W., Chanmahasathien W., Mangmool S. (2024). Adenosine A_3_ receptor: From molecular signalling to therapeutic strategies for heart diseases. Int. J. Mol. Sci..

